# Costs of HIV/AIDS treatment in Indonesia by time of treatment and stage of disease

**DOI:** 10.1186/s12913-015-1098-3

**Published:** 2015-09-30

**Authors:** Adiatma Y. M. Siregar, Noor Tromp, Dindin Komarudin, Rudi Wisaksana, Reinout van Crevel, Andre van der Ven, Rob Baltussen

**Affiliations:** Integrated Management for Prevention and Control and Treatment of HIV/AIDS (IMPACT), Bandung, Indonesia; Department of Economics, Faculty of Economics and Business, Padjadjaran University, Bandung, Indonesia; Department of Health Evidence, Radboud University Nijmegen Medical Center, Nijmegen, The Netherlands; Department of Internal Medicine, Hasan Sadikin Hospital/Faculty of Medicine, Padjadjaran University, Bandung, Indonesia; Department of Internal Medicine, Radboud University Nijmegen Medical Center, Nijmegen, The Netherlands

**Keywords:** Cost Analysis, Antiretroviral Therapy, HIV, Indonesia

## Abstract

**Background:**

We report an economic analysis of Human Immunodeficiency Virus (HIV) care and treatment in Indonesia to assess the options and limitations of costs reduction, improving access, and scaling up services.

**Methods:**

We calculated the cost of providing HIV care and treatment in a main referral hospital in West Java, Indonesia from 2008 to 2010, differentiated by initiation of treatment at different CD4 cell count levels (0–50, 50–100, 100–150, 150–200, and >200 cells/mm^3^); time of treatment; HIV care and opportunistic infections cost components; and the costs of patients for seeking and undergoing care.

**Discussion:**

Before antiretroviral treatment (ART) initiation, costs were dominated by laboratory tests (>65 %), and after initiation, by antiretroviral drugs (≥60 %). Average treatment costs per patient decreased with time on treatment (e.g. from US$580 per patient in the first 6 month to US$473 per patient in months 19–24 for those with CD4 cell counts under 50 cells/mm^3^). Higher CD4 cell counts at initiation resulted in lower laboratory and opportunistic infection treatment costs. Transportation cost dominated the costs of patients for seeking and undergoing care (>40 %).

**Conclusions:**

Costs of providing ART are highest during the early phase of treatment. Costs reductions can potentially be realized by early treatment initiation and applying alternative laboratory tests with caution. Scaling up ART at the community level in certain high prevalence settings may improve early uptake, adherence, and reduce transportation costs.

## Background

The face of the HIV/AIDS epidemic in Indonesia is changing. While the epidemic started among injecting drug users, now it is shifting towards the general population. The percentage of HIV transmission through injecting drug use decreased from 13 % in 2010 to 7 % in 2014, and heterosexual transmission increased from 31 to 52% during the same period [1].

Indonesia’s national response to the epidemic focuses on a wide range of programs, including continuing support for care and treatment programs [2]. The need for antiretroviral therapy (ART) is expected to increase from approximately 30,000 patients in 2008 to almost 87,000 patients in 2014 [3]. However, only 24 % of people living with HIV (PLHIV) who are eligible for treatment in Indonesia receive ART which indicates the need for the government to increase ART services, and puts pressure on the already-constrained budget for HIV/AIDS control in Indonesia [4]. The guideline for providing ART in Indonesia has also changed. Since 2011, the treatment was given to patients with CD4 cell count of less than 350 cells/mm^3^, with the exception of pregnant women with HIV, babies born from women with HIV, and PLHIV with TB and Hepatitis B who are not yet on ART (these PLHIV received ART regardless of their CD4 cell count) [5, 6]. In 2014 this guideline was revised. While the CD4 cell count cut- off remained the same, the list of groups of PLHIV who are eligible for ART regardless of their CD4 cell count were extended. This now includes PLHIV with Hepatitis C, children under five with HIV, high risk groups with HIV, serodiscordant couples, and PLHIV living in a region with increasing HIV epidemic [7]. In this respect, the information on costs of HIV treatment becomes even more important.

There is an urgent need to address the following research questions. First, what are the present treatment costs per person per year, by time of treatment and stage of disease, and is there any potential to reduce these costs? There is very little evidence on this question from the Indonesian context. Yet, in recent years, much evidence has become forward from African studies. For example, a recent multi-country study including 161 facilities has shown that facility-level ART costs on average $208 per patient-year across Ethiopia, Malawi, Rwanda and Zambia. Costs were higher in South Africa, at $682 [8]. Another study, using multiple independent mathematical models in four settings-South Africa, Zambia, India, and Vietnam- concluded that early initiation of ART (at CD4 levels ≤500 cells/μL) is more cost-effective than late treatment [9]. Also, there is (limited) international evidence that delivery of ART is less costly at the community level than at the hospital level [10]. The limited Indonesian data that is available suggests that the use of less intense diagnostic and treatment procedures might lead to cost savings [11–13]; however, this data has not been placed in the context of total treatment costs.

Second, what are the costs of scaling up ART? International evidence suggests that cost increases stemming from increased patient numbers can partly be offset by reduced average treatment costs as patients are treated earlier [14]; however, there is no specific evidence for Indonesia to support this hypothesis.

Third, what is the financial burden of patients accessing care? Although international studies have shown that the financial burden on patients may constitute a barrier to treatment [15] and may affect adherence and retention to treatment (unpublished data [13, 14]), available evidence from Indonesia stems from a single study only [16].

This paper presents an economic analysis of the provision of ART and care for HIV/AIDS patients from both the health care provider and patient perspectives. It presents a cost profile differentiated by initiation of treatment at different CD4 cell count levels; time on treatment; cost components (e.g., drug and personnel costs) for HIV care and opportunistic infections (OIs); and finally patient costs of seeking and undergoing care. We believe our approach is unique in the context of Indonesia and Asia and can be generalized to other settings with similar HIV/AIDS epidemics and health system profiles.

## Methods

### Study setting and study population

The study was conducted in Bandung, at an HIV/AIDS clinic in the largest public referral and teaching hospital (Hasan Sadikin) in West Java province (43 million inhabitants). The clinic is visited by high risk groups and the general population, and delivers HIV-related services such as voluntary counseling and testing, ART, and sexually transmitted infections services. The clinic operates at full capacity because it is among the few clinics that deliver ART in Bandung. The clinic generates its own revenues through government, hospital, and private funding; ART-related services are free, except hospitalization and the registration fee.

The study included all records on inpatients and outpatients starting ART in the clinic between 2008 and 2010. The study population was divided into five groups by CD4 cell count: 0–50, 50–100, 100–150, 150–200, and >200 cells/mm^3^. The starting point of every patient initiating ART was uniformed as month 1, and patients were analyzed at 6, 12, 18, and 24 months on ART. We also observed the pre-ART period when patients received HIV care and treatment but had not yet initiated ART, which ranged from 1 week to 6 months before treatment initiation. Based on these starting points, each patient’s treatment pattern was tracked from available medical and financial records and all costs were calculated. Patients were required to visit the clinic monthly to take their antiretroviral drugs (ARVs), and undergo CD4 cell count, viral load, and routine laboratory tests approximately every 6 months.

### Data collection and cost estimation

The cost estimation was divided into health care costs (costs related to the consumption of resources in the health care system) and patient costs (costs falling on the patient for seeking and undergoing care). Health care costs were divided into hospitalization, outpatient visits, OI treatment, and ARVs, as well as CD4 cell count, viral load, and routine laboratory test costs. The micro-costing approach [17] was used to calculate the unit cost of an outpatient visit and OI treatment. All resources consumed, prices, and salaries related to service utilization were listed and estimated based on clinical records, pharmacy databases, staff interviews, government price standards, and hospital or market prices.

Outpatient costs were calculated based on the total recurrent and capital costs of the clinic. Recurrent personnel costs were estimated based on actual wages or government salary scales [18]. Other recurrent costs (e.g., administration goods consumed during the observation period) were estimated using both actual and market prices. Capital costs included trainings and workshops attended by the clinic staff, and unit costs for organizing these activities were obtained from the actual budgetary or governmental records. Market prices were used to estimate other capital costs, including equipment, furniture, and start-up costs (e.g., renovation costs, if applicable). Capital costs were subsequently annualized on the basis of the lifetime of the capital items, using a 3 % discount rate [17]. We omitted the cost of utilities (i.e., water and electricity). This result was then multiplied by the proportion of time allocated by the clinic to deliver ART, calculated through a separate time motion study in which we observed (in a week time, within the clinic) all clinical activities and calculated the amount of time spent on ART-related duties per week by the clinic staff. The total outpatient visit cost was then divided by the number of total outpatient visits to obtain the unit cost per outpatient visit. Patients registered as outpatient were never also registered as inpatient in the same period. For example, a patient who is registered as inpatient in a certain month may be registered as outpatient in the next month, but never at the same month. The details of the outpatient cost are presented in the Appendix: Table 6).

The OI treatment costs were calculated based on the medical resources consumed by OI treatment (e.g., drugs and equipment), excluding hospitalization. Medical records and the physician’s patient database were used to estimate resource utilization, and the official hospital prices issued in 2011 were used to calculate the unit costs of drugs and equipment. We obtained the unit cost of OI treatment by dividing the total cost of OI treatment for each CD4 cell count group by its population. We were unable to retrieve data regarding the specific OIs that drugs and equipment were used to treat. The average unit cost of OI treatment is presented in Appendix: Table 7).

Because the data was limited, we did not perform micro-costing when calculating the costs of hospitalization, ARVs, or laboratory tests. We used the World Health Organization’s Choosing Interventions that are Cost Effective (WHO-CHOICE) estimates [19] to estimate the per day inpatient hotel cost, which we then used to calculate the total hospitalization cost. The WHO-CHOICE estimates for inpatient cost include items such as personnel, capital, and food costs, and exclude drugs and diagnostic test costs. The prices of ARV drugs (except for Tenofovir) issued by *Kimia Farma* (a national pharmaceutical corporation) were used as the unit costs of ARVs, while the price of Tenofovir was based on Bender et al. [20]. The unit costs of laboratory tests (CD4 cell count, viral load, and other laboratory tests) were derived from the 2011 official hospital price for each test. The summary of all unit costs used is presented in the Appendix: Table 8).

The patient costs were estimated by conducting a survey among 41 patients undergoing ART at the hospital. We collected information including (but not limited to) clinic service fee, travel costs, travelling time, the average number of daily working hours, and monthly expenditures. Based on this information, first we calculated the amount of productive time per patient (in minutes), basically total time spent at work per month in minutes. Second, we calculated patient productivity per minute (i.e. monthly expenditure per minute as a proxy for income/productivity per minute). Third, we estimated the total productivity loss per patient as the amount of minutes spent to undergo the treatment (i.e. time spent for traveling, queueing/waiting, and treatment) times patient productivity per minute. Patients did not have to pay for ARV, ARV monitoring, other lab tests, or OI medication/treatment. To avoid double counting in calculating treatment costs from the societal perspective, we exempted the clinic service fee from this specific calculation (but it is included in the patient cost calculation).

All costs were measured in Rupiah, and converted to US$ using the 2010 exchange rate [21]. Both the utilization and cost data were analyzed using Microsoft Excel 2007. We report the costs from both health care system and patient perspectives, and performed statistical tests to find out the significance of any costs differences between period and CD4 cell count level.

Secondary data related to patients (e.g. ARV and OI drugs intake per patient) were taken from the clinic’s patients database (in the form of an Excel file). The database was anonymized prior to analysis (we utilize the patients hospital ID number during analysis), and none of the patients personal identity was published in any part of the study. On the event of patient costs (primary) data collection, all patients were asked to fill in written informed consent forms prior to participating in the survey and the survey was anonymous (no patient names were collected). The survey was conducted by a group of enumerators and authors only received the results. The study was approved by the Padjadjaran University Indonesia, Medical Faculty ethical committee.

## Results

### Patient characteristics

Patient characteristics are presented in Table 1. The majority of patients within the CD4 cell count of 0 – 150 cells/mm^3^ group are males (151 male, 34 female), while females dominate the >150 cells/mm^3^ group (11 male, 16 female). In average, there are more males across the CD4 cell count groups (72 %). The average age of patients across the groups is 30 years old, and the majority are married, employed, and have experience with injecting drug use. The highest education level attained by patients was the secondary level (high school).

**Table 1:** Characteristics of patients on ART

		CD4 cell count at time of starting ART					
		0–50	50–100	100–150	150–200	>200	Overall
No. of observation at the start of ART		96	33	22	17	10	178
CD4 level at the start of ART , mean^a^		19 (16 – 22)	71 (66 – 76)	124 (118 – 130)	177 (170–184)	275 (252 – 298)	39 (13–110)^b^
Sex (male)		80 %	82 %	59 %	41 %	40 %	72 %
Age, mean^a^		30 (29 - 31)	30 (28–32)	29 (28–30)	27 (26–28)	27 (25 – 29)	30 (29–31)
History of injecting drug use		69 %	70 %	55 %	41 %	70 %	65 %
Marital status	Married	52 %	58 %	45 %	35 %	60 %	51 %
	Not married	36 %	24 %	41 %	29 %	10 %	33 %
	Widowed/ divorced	10 %	15 %	14 %	29 %	30 %	15 %
Occupation status	Employed	67 %	82 %	86 %	82 %	80 %	74 %
	Student	2 %	-	-	6 %	-	2 %
	Unemployed	30 %	15 %	14 %	12 %	20 %	23 %
Highest education	Primary	3 %	-	-	-	-	2 %
	Secondary	58%	51 %	59 %	77 %	80 %	60 %
	Tertiary	36 %	48 %	41 %	24 %	20 %	37 %

^a^95 % CI, ^b^Median (IQR)

### Resource utilization and costs of providing ART

Table 2 presents the resources used to provide ART. Hospitalization occurred only before ART and up to 6 months after treatment was initiated; the duration ranged from 3 to 20 days. The switch to second line ART occurred in 5 % of patients with a CD4 cell count of 0 − 50 cells/mm^3^ and 15 % of those with 50 − 100 cells/mm^3^. Few patients with CD4 cell counts >200 cells/mm^3^ were hospitalized and received OI treatment. Details regarding unit costs per item are summarized in the Appendix: Table 8).

**Table 2:** Resource utilization of patients on ART by CD4 cell count at the start of ART, per specified period

CD4 cell count	Item	Period					Average 1–24 months
		Before ART	1–6 months	7–12 months	13–18 months	19–24 months	
0–50	Number of patients	96	96	95	84	61	
	% hospitalized^a^	14 %	22 %	-	-	-	
	Average days of hospitalization^b^	6 (4–8)	15 (10–20)	-	-	-	0.2^c^
	Number of outpatient visit	95	47	75	63	41	3^c^
	% of patients treated for OI^a^	2 %	63 %	27 %	18 %	8 %	29 %
	% switched to 2^nd^ line ARV^a^	-	-	1 %	2 %	3 %	2 %
	Number of CD4 tests	95	47	75	63	41	3^c^
	Number of viral load tests	-	6	16	9	4	0.4^c^
	Number of routine lab tests	87	45	74	62	40	3^c^
50–100	Number of patients	33	33	33	32	25	
	% hospitalized^a^	3 %	12 %	-	-	-	
	Average days of hospitalization^b^	3	6 (5–7)	-	-	-	0.2^c^
	Number of outpatient visit	36	13	29	20	10	2^c^
	% of patients treated for OI^a^	3 %	36 %	21 %	13 %	4 %	19 %
	% switched to 2^nd^ line ARV^a^	-	3 %	3 %	6 %	12 %	7 %
	Number of CD4 tests	36	13	29	20	10	2^c^
	Number of viral load tests	-	5	6	4	-	0.5^c^
	Number of routine lab tests	30	14	28	20	9	2^c^
100–150	Number of patients	22	22	22	18	13	
	% hospitalized^a^	-	14 %	-	-	-	
	Average days of hospitalization^b^	-	7	-	-	-	0.3^c^
	Number of outpatient visit	29	8	18	13	7	2^c^
	% of patients treated for OI^a^	-	45 %	18 %	11 %	15 %	22 %
	% switched to 2^nd^ line ARV^a^	-	-	-	-	-	
	Number of CD4 tests	29	8	18	13	7	2^c^
	Number of viral load tests	1	1	4	3	-	0.4^c^
	Number of routine lab tests	21	8	17	13	7	2^c^
150–200	Number of patients	16	16	13	13	11	
	% hospitalized^a^	12 %	6 %	-	-	-	
	Average days of hospitalization^b^	5	3	-	-	-	0.2^c^
	Number of outpatient visit	26	9	9	11	7	3^c^
	% of patients treated for OI^a^	-	47 %	14 %	21 %	-	27 %
	% switched to 2^nd^ line ARV^a^	-	-	-	-	-	
	Number of CD4 tests	26	9	9	11	7	3^c^
	Number of viral load tests	1	4	4	1	-	0.6^c^
	Number of routine lab tests	16	8	9	11	7	3^c^
>200	Number of patients	10	10	10	7	4	
	% hospitalized^a^	-	-	-	-	-	
	Average days of hospitalization^b^	-	-	-	-	-	
	Number of outpatient visit	17	5	6	4	4	3^c^
	% of patients treated for OI^a^	-	10 %	-	-	-	10 %
	% switched to 2^nd^ line ARV^a^	-	-	-	-	-	
	Number of CD4 tests	17	5	6	4	4	3^c^
	Number of viral load tests	-	-	-	-	-	
	Number of routine lab tests	9	5	6	4	4	3^c^

^a^for the whole sample within the indicated period,^b^ 95 CI%, ^c^per patient

**Fig. 1:**
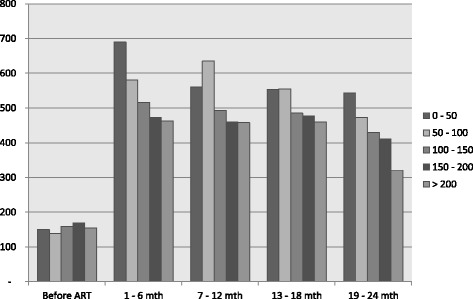
Average service costs per patient per specified period, health care system perspective (US$). This figure presents the average service costs per patient taking ART. The average costs are separated into specific periods, namely before ART, 1–6 months, 7–12 months, 13–18 months, and 19–24 months within ART. These costs are further separated into CD4 cell count group, namely 0–50, 50–100, 100–150, 150 - 200, and >200 cells/mm^3^. The figure shows how the average costs per patient in different CD4 cell count groups relatively decrease after the start of ART

**Fig. 2:**
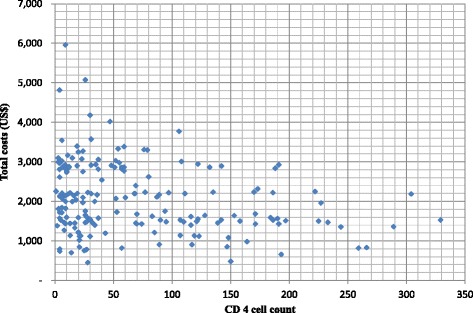
Appendix (Figure A). HIV treatment total costs per patient on ART, over 24 months. This figure shows the scatter diagram of total costs of patients undertaking HIV treatment over 24 months. The patients within the group of 0–50 cells/mm^3^ CD4 cell count have the largest variance in costs (e.g. patients with highest or lowest average treatment costs are within this group)

Table 3 details the costs associated with providing ART. Before ART initiation, costs were mainly dominated by laboratory tests (including the CD4, viral load, and routine laboratory tests). After the initiation of ART, costs were dominated by ARV, regardless of patients’ CD4 levels. Both total costs and per patient average costs decreased over time after ART initiation. The one anomaly was the OI drugs/treatment cost for patients with a CD4 level of 50 − 100 cells/mm^3^, which increased from US$725 in 1–6 months to US$2099 in 7–12 months. A relatively high CD4 cell count at treatment initiation relates to relatively low costs of ARVs, laboratory tests, and OI drugs/treatment. Figure 1 shows the average costs per patient for different CD4 cell count levels and over time. The highest average costs for 24 months of ART per patient were for patients with a CD4 cell count <50 cells/mm^3^. The distribution of cost is provided in the Appendix: Figure 2). The average costs difference between patients undergoing the first 6 months of treatment and the 24 months of treatment is statistically significant within the group of patients with CD4 cell count < 50 cells/mm^3^, 50 – 100 cells/mm^3^, and those with >200 cells/mm^3^. The average 2 year treatment costs difference between the patients with CD4 cell count < 50 cells/mm^3^ and the groups with higher CD4 cell count is also statistically significant, except with patients with CD4 cell count between 100 – 150 cells/mm^3^. The statistical significance test is summarized in Appendix: Table 9.

**Table 3:** Health care costs of patients on ART by CD4 cell count at the start of ART, per specified period (US$^a^)

CD4 cell count	Item	Period					Average 1–24 months per patient
		Before ART	1–6 months	7–12 months	13–18 months	19–24 months	
0–50	Number of patients	96	96	95	84	61	
	Hospitalization	1483 (10 %)	6161 (9 %)	-	-	-	64
	Outpatient visits	1110 (8 %)	6707 (10 %)	6462 (12 %)	5247 (11 %)	3517 (11 %)	258
	OI treatment	86 (1 %)	10867 (16 %)	2163 (4 %)	564 (1 %)	1538 (5 %)	168
	ARV drugs	-	40012 (60 %)	41281 (78 %)	37724 (81 %)	26915 (81 %)	1742
	CD4 test	1254 (9 %)	620 (1 %)	990 (2 %)	832 (2 %)	541 (2 %)	36
	Viral load test	-	396 (1 %)	1056 (2 %)	594 (1 %)	264 (1 %)	27
	Routine lab test	10452 (73 %)	1488 (2 %)	1289 (2 %)	1548 (3 %)	363 (1 %)	53
	Total costs	14377 (100 %)	66205 (100 %)	53196 (100 %)^c^	46472 (100 %^c^	33115 (100 %)^c^	2346
	Average costs per patient^b^	150 (139 – 160)	690 (593–787)	560 (521–599)	553 (515–592)	543 (480–606)	
50–100	Number of patients	33	33	33	32	25	
	Hospitalization	57 (1 %)	418 (2 %)	-	-	-	13
	Outpatient visits	421 (9 %)	2337 (12 %)	2232 (11 %)	1951 (11 %)	1110 (11 %)	244
	OI treatment	12 (0.3 %)	725 (4 %)	2099 (10 %)	13 (0.1 %)	9 (0.1 %)	86
	ARV drugs	-	14694 (77 %)	15360 (73 %)	14763 (83 %)	9058 (87 %)	1734
	CD4 test	475 (10 %)	172 (1 %)	383 (2 %)	264 (1 %)	132 (1 %)	30
	Viral load test	-	330 (2 %)	396 (2 %)	264 (1 %)	-	30
	Routine lab test	3608 (79 %)	478 (2 %)	494 (2 %)	508 (3 %)	104 (1 %)	49
	Total costs	4570 (100 %)	19138 (100 %)	20947 (100 %)	17749 (100 %)	10405 (100 %)^c^	2186
	Average costs per patient^b^	138 (126–151)	580 (567–593)	635 (622–648)	555 (542–568)	473 (460–486)	
100–150	Number of patients	22	22	22	18	13	
	Hospitalization	-	418 (4 %)	-	-	-	19
	Outpatient visits	339 (10 %)	1566 (14 %)	1391 (13 %)	1157 (13 %)	771 (14 %)	258
	OI treatment	-	815 (7 %)	77 (0.7 %)	29 (0.3 %)	2 (0.04 %)	42
	ARV drugs	-	8147 (72 %)	8587 (79 %)	6858 (79 %)	4661 (83 %)	1500
	CD4 test	383 (11 %)	106 (1 %)	238 (2 %)	172 (2 %)	92 (2 %)	32
	Viral load test	66 (2 %)	66 (1 %)	264 (2 %)	198 (2 %)	-	26
	Routine lab test	2547 (76 %)	222 (2 %)	293 (3 %)	317 (4 %)	63 (1 %)	46
	Total costs	3333 (100 %)	11329 (100 %)	10839 (100 %)	8722 (100 %)	5585 (100 %)	1922
	Average costs per patient^b^	159 (144–173)	515 (500–529)	493 (478–507)	485 (470–499)	430 (415–444)	
150–200	Number of patients	17	17	14	14	11	
	Hospitalization	190 (7 %)	57 (1 %)	-	-	-	3
	Outpatient visits	304 (11 %)	1133 (14 %)	982 (15 %)	935 (14 %)	654 (14 %)	263
	OI treatment	-	66 (1 %)	3 (0.05 %)	5 (0.1 %)	-	4
	ARV drugs	-	6188 (77 %)	4865 (76 %)	5162 (77 %)	3717 (82 %)	1418
	CD4 test	343 (12 %)	119 (1 %)	119 (2 %)	145 (2 %)	92 (2 %)	34
	Viral load test	66 (2 %)	264 (3 %)	264 (4 %)	66 (1 %)	-	39
	Routine lab test	1969 (69 %)	200 (2 %)	195 (3 %)	362 (5 %)	63 (1 %)	57
	Total costs	2870 (100 %)	8020 (100 %)	6421 (100 %)	6670 (100 %)	4522 (100 %)	1818
	Average costs per patient^b^	169 (136–201)	472 (445–499)	459 (432–485)	476 (450–503)	411 (384–438)	
>200	Number of patients	10	10	10	7	4	
	Hospitalization	-	-	-	-	-	
	Outpatient visits	199 (13 %)	701 (15 %)	608 (13 %)	363 (12 %)	257 (20 %)	247
	OI treatment	-	1 (0.02 %)	-	-	-	0.1
	ARV drugs	-	3637 (79 %)	3797 (83 %)	2715 (84 %)	938 (73 %)	1366
	CD4 test	224 (15 %)	66 (1 %)	79 (2 %)	53 (2 %)	53 (4 %)	35
	Viral load test	-	-	-	-	-	
	Routine lab test	1119 (73 %)	223 (5 %)	92 (2 %)	68 (2 %)	35 (3 %)	50
	Total costs	1541 (100 %)	4623 (100 %)	4572 (100 %)	3218 (100 %)	1281 (100 %)^c^	1699
	Average costs per patient^b^	154 (114–195)	462 (437–487)	457 (432–482)	460 (435–485)	320 (295–345)	

^a^except for number of patients, ^b^95 % CI, ^c^difference is significant between first 6 months and the period measured, 95 % CI

### Patient costs per visit

Table 4 presents patient costs. The average patient costs per visit are US$10 and US$11, for patients with CD4 cell counts below and above 200 cells/mm^3^, respectively. Transportation cost and the clinic fee dominated the costs, while productivity loss accounted for less than 25 % of the total cost per visit. Per visit, almost all patients spent US$2 for the registration fee and US$5 − US$6 for transportation. The mean time to reach the clinic was approximately 1 h (most patients lived < 20 km away) and the average time spent in the clinic was approximately 100 min. There were no major differences in patient costs between patients with CD4 cell counts less than or greater than 200 cells/mm^3^ (we did not perform significance test for this due to our small sample size). Most patients in our survey travelled using either public transport (54 %) or motorcycle (42 %).

**Table 4:** Patient costs of accessing ART (patient’s perspective) (US$)

CD4 level at the start of ART		Estimated average per visit access costs			Monthly household expenditure
	Average transportation cost	Average estimated productivity loss	Average fee for clinic	Average per visit total costs	
0 – 200 (*n* = 17)^a,b^	5.9 (0–12.2)	1.1 (0.5–1.7)	2.4 (2.0–2.7)	9.6 (2.9 – 16.3)	71.4 (41.9 – 101.0)
>200 (*n* = 23)^a,b^	4.7 (2.4–7.1)	3.2 (1.8–4.6)	2.2	11.0 (3.7 – 18.3)	147 (72 – 221)

^a^average, ^b^95 % CI

## Discussion

To our knowledge, this study is the first in Indonesia and among the few in Asia [22, 23] to estimate the cost of providing ART. The overall cost profile shows that the total costs and average costs per patient are reduced when patients’ CD4 levels are higher at the time of clinic enrollment and ART initiation. In most instances, hospitalization, OI treatment, and ART average costs per patient decrease with longer use of ART. During the early phase of the treatment, the highest costs are the costs of hospitalization, OI treatment, and ART initiation; these costs decrease over time as a result of patients’ improved health. This trend is comparable with the results of a study conducted in Southern Africa [24].

These findings lead to several observations in response to the research questions. Regarding the cost of treatment and potential cost reduction, the study confirms the hypothesis that the following measures have potential to reduce cost in ART delivery:

1. Early ART initiation. Most cost items are lower when patients’ CD4 cell counts are higher at ART initiation and especially the total cost share of hospitalization and OI treatment was reduced. CD4 cell counts can predict the likelihood of OIs; patients with CD4 cell counts >200 cells/mm^3^appear to be at lower risk for the majority of OIs, compared with patients with <200 cells/mm^3^ [25–28] and this explains our study results. Early treatment is also cost effective in resource-limited settings as well as on the global epidemic setting [9, 29, 30]. Additionally, the NIAID START Trial shows that early ART protects the health of PLHIV [31]. WHO is now preparing for a new ARV provision guideline in which it may recommend an even earlier ART initiation compared to its 2013 guideline [32]. Thus, our recommendation is strengthened by the WHO discussion and it warrants further attention. The costs implication of prolonged treatment, however, should be further studied to determine whether the costs reduction and long term costs saving resulting from early treatment offset the costs of prolonged treatment.
2. Alternative diagnostics. Before ART initiation, costs are dominated by laboratory tests (CD4, viral load, and routine laboratory tests) followed by outpatient visits and hospitalization. After ART initiation, costs were dominated by ARV drugs followed by laboratory tests. Although costs reductions in hospitalization and ARV use might be difficult to realize, reductions are possible for laboratory test costs, as is found in the DART trial in four African countries [33], which showed that ART can be delivered safely without routine laboratory monitoring for toxicity. A study within the same hospital clinic with our study demonstrated that the total lymphocyte count (TLC) is a good alternative for CD4 cell count as it is much cheaper and easier to implement in rural settings. Combining TLC test results with an algorithm of simple patient characteristics could save US$14 per patient compared with the current scenario [11]. Also, De Jong et al.[12] in Indonesia (study conducted at the same clinic as our study) and Kumarasamy et al. [34] in India found that TLC may reduce the need for routine CD4 measurements during ART (excepting the first year of treatment). In more recent studies, however, it is found that TLC may not be a reliable predictor for CD4 cell count in HIV-infected individuals in certain settings [35, 36], while it is reliable in others [37–41]. Therefore, caution is needed in applying TLC test as a replacement for CD4 test as it seems like the success of its application is varied, depending much on the specific settings and population in which it is applied. In Indonesia, another method to reduce laboratory costs is proposed by Indrati et al. [13], who found that a dual-test or single rapid-test algorithm (instead of a serial three-test algorithm) may be just as accurate and more cost-effective, although the single rapid-test should be interpreted carefully. Although these alternative laboratory testing methods may lead to costs reductions, more research is needed to determine the potential cost savings.

Regarding our second research question about scaling up ART, the study indicates that although increased ART coverage may cause a large increase in health expenditure in the short run [23, 42], it could potentially save costs in the long run. By reaching more people in need of ART, assuming that these are detected at earlier stages, costs related to opportunistic infections and hospitalization may be avoided as has been shown in our study. Importantly, providing ART can also act as HIV/AIDS prevention [43, 44] because ART treatment reduces transmission rates. Universal voluntary HIV testing and early ART could therefore have a major effect on the HIV/AIDS epidemic and could be cost saving [29, 45]. The costs of treatment of new HIV infections will be averted and may potentially free resources to prevent even more infections [14]. Considering these findings, we suggest further study regarding advantages (e.g., health benefits of early treatment) and disadvantages (e.g., budget impact) of scaling up ART in Indonesia from both the short run and long run perspectives.

In terms of the location for scaling up ART, we cannot draw strong conclusions on the basis of the costing analysis in the hospital only. Yet, there seem to be advantages in scaling up ART at the community level as this may potentially increase early detection and reduce the burden in hospital clinics [46]. Also, the shorter waiting and travel time to the clinic may lead to lower patient’s costs and better uptake and adherence of ART [46–49]. In this scenario, the hospital and community health centres will have different roles (Table 5). The hospital will be a referral centre for complicated AIDS cases and treatment of OIs just as current practice [46, 50]. Because patients become relatively stable over time (indicated by decrease in hospitalization and OI treatment over time) they could continue ART at community clinics, reducing the hospital burden. Patients that initiate ART at >200 cell/mm^3^ could also obtain ART at the community health care center, as our analysis suggests that hospitalization and OIs are rare in this population. In addition, patients mostly utilize first line ARV, and no patients with CD4 cell counts >100 cells/mm^3^ switch to second line ARV (Table 2), indicating a low rate of treatment failure within this group [51]. As such, the ARV distribution (in terms of medicine type) in community/primary health care centres for patients with CD4 cell counts >100 cells/mm^3^ might not be too complex, as most patients are likely to require only first line ARV. Currently, there are only two primary health care centers in Bandung that provide ART, which presents considerable potential to increase the service to other community clinics.

**Table 5:** Recommendation on role of clinics in delivering ART^a^

Costs Items	Type of Clinic	
	Hospital	Community/Primary Health Care Centre
Hospitalization	+	-
Outpatient visits	+/-	+
OI treatment	+^b^	+
ARV drugs	+/-	+
CD4 test	+	-
Viral load test	+^c^	-
Routine lab test	+	+/-

*ART* antiretroviral treatment

^a^‘ + ’ and ‘-’ denote respectively a role of high and low importance for the clinic in the specified activities in HIV/AIDS control

^b^for severe cases, ^c^ if necessary

However, providing ART at all community health clinics in Indonesia at this stage seems inefficient due to the low HIV prevalence in the general population which will result in a low patient load per clinic for which all community staff will require training [52]. Therefore, providing HIV services through clinics in certain high prevalence settings such as prisons or cities may be preferable [53, 54], although this strategy requires further study.

Regarding our third research question about patients’ financial burden, the study shows that patient costs per visit are US$10 and US$11, for patients with CD4 cell counts below and above 200 cells/mm^3^. This relates to approximately 14 and 7 % of their monthly expenditure (a proxy of monthly income), respectively. Especially for patients with CD4 cell counts <200 cells/mm^3^ these costs could be a barrier, as it exceeds 10 % of their monthly expenditure and can be considered to be catastrophic for a household economy [55].

Transportation comprises the highest proportion of costs: 62 and 43 % for patients with CD4 cell counts below and above 200 cells/mm^3^, respectively, and this is comparable with the finding of Riyarto et al. [16] in Indonesia. A study by Haroen et al. [49] in Bandung, Indonesia, and international studies by Portelli et al. [47], Brinkhof et al. [48], and Posse et al. [15] have shown that transportation costs are a common reason why patients cease ARV. This information provides another reason to scale up ART at community level, as it likely reduces transportation costs for patients and may increase the uptake of ART, especially of patients with CD4 cell counts <200 cells/mm^3^.

### Study limitations

Our results should be interpreted with some caution. First, this study has evaluated a contextualized ART service delivery model, which may hamper the generalizability of its results. Cost structures and levels as well as patient populations are likely to vary between clinics, and specific costing studies for other settings (e.g., other hospitals, community/primary health centers, and prisons) should be considered. Caution should be exercised when interpreting our result in other resource limited settings. Second, we may have overestimated the total patient costs of seeking and undergoing care as this was based on assumptions regarding patients’ labour productivity losses, and not on empirical data collection on these losses per se. Third, although we have conducted a time motion study to control for inefficiency in personnel performance and equipment use related to ART delivery in the clinic, discrepancies may still exist, and we may have over- or undervalued the total costs. Fourth, we did not perform any comparison between WHO CHOICE estimates (that we used for calculating inpatient cost) and any local data. Although this is an important aspect, currently there is very limited local data available to do this comparison. Fifth, it is important to note that the unit costs and prices data that we used are from year 2010–2012, depending on availability. As such, we believe these unit costs have changed overtime and our results should be interpreted with this note in mind (e.g. ARV drugs prices may have decreased since government are producing more ARV locally [2], patients monthly expenditure has been rising due to inflation).

## Conclusion

Three main conclusions can be derived from our study. First, we show that the costs of providing ART are highest during the early phase of treatment, and will decrease and stabilize as treatment progresses. Second, our findings suggest that costs reduction can be potentially realized by early treatment initiation (which may reduce hospitalization, OI drug/treatment costs, and patient mortality) and by applying alternative laboratory tests with caution. Third, scaling up ART at the community level in certain high prevalence settings has potential to save costs and improve uptake and adherence. However, provision of ART at all community clinics seems inefficient due to the low prevalence in the general population and options to select certain clinics in high prevalence areas need further investigation.

## Appendix

**Table 6:** Yearly Cost of ART outpatient service (US$)

Type of Cost	US$ (2010)	Percent
1. CAPITAL COST (Annualized)		
1.1 Personnel (trainings & workshops)	111.37	0.5
1.2 Building/Space	477.24	2.0
1.3 Equipments	510.72	2.1
*Subtotal*	*1099.33*	
2. RECURRENT COST		
2.1 Personnel (number)	21123.40	91.7
Medical doctor (4)	6701.66	
Nurse (2)	5370.67	
Laboratory (1)	1196.22	
Administration and cleaning service (6)	15178.81	
2.2 Supplies	895.13	3.7
*Subtotal*	*23018.54*	
T o t a l	24117.86	
Unit cost per visit	11.69

**Table 7:** Average unit costs of OI treatment by CD4 cell count at the beginning of ART (US$)

CD4 cell count	Period				
	Before ART^a^	1–6 months^a^	7–12 months^a^	13–18 months^a^	19–24 months^a^
0–50	1 (0–2)	113 (48–179)	23 (3–42)	7 (0–16)	25 (0–60)
50–100	0.4 (0–1)	22 (1–43)	64 (0–170)	0.4 (0–1)	0.3 (0–1)
100–150	-	37 (0–94)	3 (0.1–7)	2 (0–3)	0.2 (0–0.4)
150–200	-	4 (0–9)	0.2 (0–1)	0.4 (0–1)	-
>200	-	0.1 (0–0.3)	-	-	-

^a^95 % CI

**Table 8:** Unit Costs per Cost Item (US$)

Costs Item	Unit Cost	Source
Inpatient day	19.2	WHO CHOICE
Outpatient visit	11.7	Own calculation
Monthly average unit costs of ARV drugs (min-max)		
First line	28 (7–79)	*Kimia Farma*
Second line	90 (56–117)	*Kimia Farma* and Bender *et al.,* [20]
CD4 test	13.2	Hospital Decree
Viral load test	66	Hospital Decree
Average unit costs of laboratory tests (confidence interval)	6 (4–8)	Hospital Decree

**Table 9:** Mean one way t-test result (P-value), 95 % CI

Mean t-test for unit cost per 2 years per patient between CD4 cell count group				
Lowest CD4 cell count group	CD4 cell count			
	51–100	101–150	151–200	>200
0–50	0.49	0.03	0.004	0.0006
Mean t-test for unit cost per 6 months per patient between period by CD4 cell count group				
CD4 cell count in 0–6 months	Period (months)			
	7–12	13–18	19–24	
0–50	0.008	0.006	0.007	
51–100	0.20	0.30	0.03	
101–150	0.40	0.36	0.19	
151–200	0.42	0.47	0.17	
>200	0.47	0.49	0.01	
